# A Recombinant Vaccine Effectively Induces C5a-Specific Neutralizing Antibodies and Prevents Arthritis

**DOI:** 10.1371/journal.pone.0013511

**Published:** 2010-10-20

**Authors:** Kutty Selva Nandakumar, Åsa Jansson, Bingze Xu, Niclas Rydell, Anna M. Blom, Rikard Holmdahl

**Affiliations:** 1 Medical Inflammation Research, Department of Experimental Medicine, Lund University, Lund, Sweden; 2 Medical Inflammation Research, Department of Medical Biochemistry and Biophysics, Karolinska Institutet, Stockholm, Sweden; 3 Resistentia Pharmaceuticals AB, Uppsala, Sweden; 4 Section of Medical Protein Chemistry, Department of Laboratory Medicine, Lund University, Skåne University Hospital, Malmö, Sweden; University of Pittsburgh, United States of America

## Abstract

**Objectives:**

To develop and validate a recombinant vaccine to attenuate inflammation in arthritis by sustained neutralization of the anaphylatoxin C5a.

**Methods:**

We constructed and expressed fusion protein of C5a and maltose binding protein. Efficacy of specific C5a neutralization was tested using the fusion protein as vaccine in three different arthritis mouse models: collagen induced arthritis (CIA), chronic relapsing CIA and collagen antibody induced arthritis (CAIA). Levels of anti-C5a antibodies and anti-collagen type II were measured by ELISA. C5a neutralization assay was done using a rat basophilic leukemia cell-line transfected with the human C5aR. Complement activity was determined using a hemolytic assay and joint morphology was assessed by histology.

**Results:**

Vaccination of mice with MBP-C5a led to significant reduction of arthritis incidence and severity but not anti-collagen antibody synthesis. Histology of the MBP-C5a and control (MBP or PBS) vaccinated mice paws confirmed the vaccination effect. Sera from the vaccinated mice developed C5a-specific neutralizing antibodies, however C5 activation and formation of the membrane attack complex by C5b were not significantly altered.

**Conclusions:**

Exploitation of host immune response to generate sustained C5a neutralizing antibodies without significantly compromising C5/C5b activity is a useful strategy for developing an effective vaccine for antibody mediated and C5a dependent inflammatory diseases. Further developing of such a therapeutic vaccine would be more optimal and cost effective to attenuate inflammation without affecting host immunity.

## Introduction

Complement is important for host defense but its inappropriate activation can result in tissue injury and damage. Upon cleavage, C3 and C5 release C3a and C5a fragments that are potent anaphylatoxins and leukocyte chemoattractants capable of stimulating and modulating inflammatory responses [1]. Anaphylatoxins are implicated in the pathogenesis of several diseases including allergy, autoimmunity, neurodegenerative diseases and cancer [2], [3] but could also play a protective role against certain infections [4]. On the other hand, C5b represents the initial molecule of the terminal complement pathway that play an essential role in the protection against infectious diseases [5] and in antigen induced arthritis [6]. Activation of complement results in the cleavage of C3 leading to C5 activation [7], but C5a can be generated in the absence of C3 as well [8]. C5a thus generated is involved in recruitement and activation of inflammatory cells [9], which can not only regulate adaptive immune responses [10], [11] but also exhibit anti-inflammatory properties [12]. Since C5 is essential for immunological functions [5], neutralization of C5a without affecting the essential function of C5b (formation of MAC) becomes important [13]. Especially, sustained neutralization of C5a by exploitation of host immunity will be more optimal and cost effective for therapeutics.

Development of rheumatoid arthritis (RA) involves a cascade of inflammatory events leading to joint and cartilage erosions. Autoantibodies prevalent in RA might play an important role in the disease development and most widely used animal models are dependent on antibody-mediated pathologies [14]–[17]. Antibodies in the form of immune complexes might play a central role in triggering inflammatory pathways in the joint [18], especially C5a binding to these immune complexes can attract granulocytes to the articular cartilage that can release inflammatory mediators (proteases, cytokines, chemokines, and reactive oxygen and nitrogen radicals) perpetuating inflammation and autoimmunity. In the present study, breaking tolerance towards C5a by vaccination to induce polyclonal anti-C5a response, C5a/C5b neutralizing capacity of the induced antibodies and their effect on arthritis development in various mouse models were assessed.

## Results

### Effect of C5a vaccination on CIA

**Figure 1:**
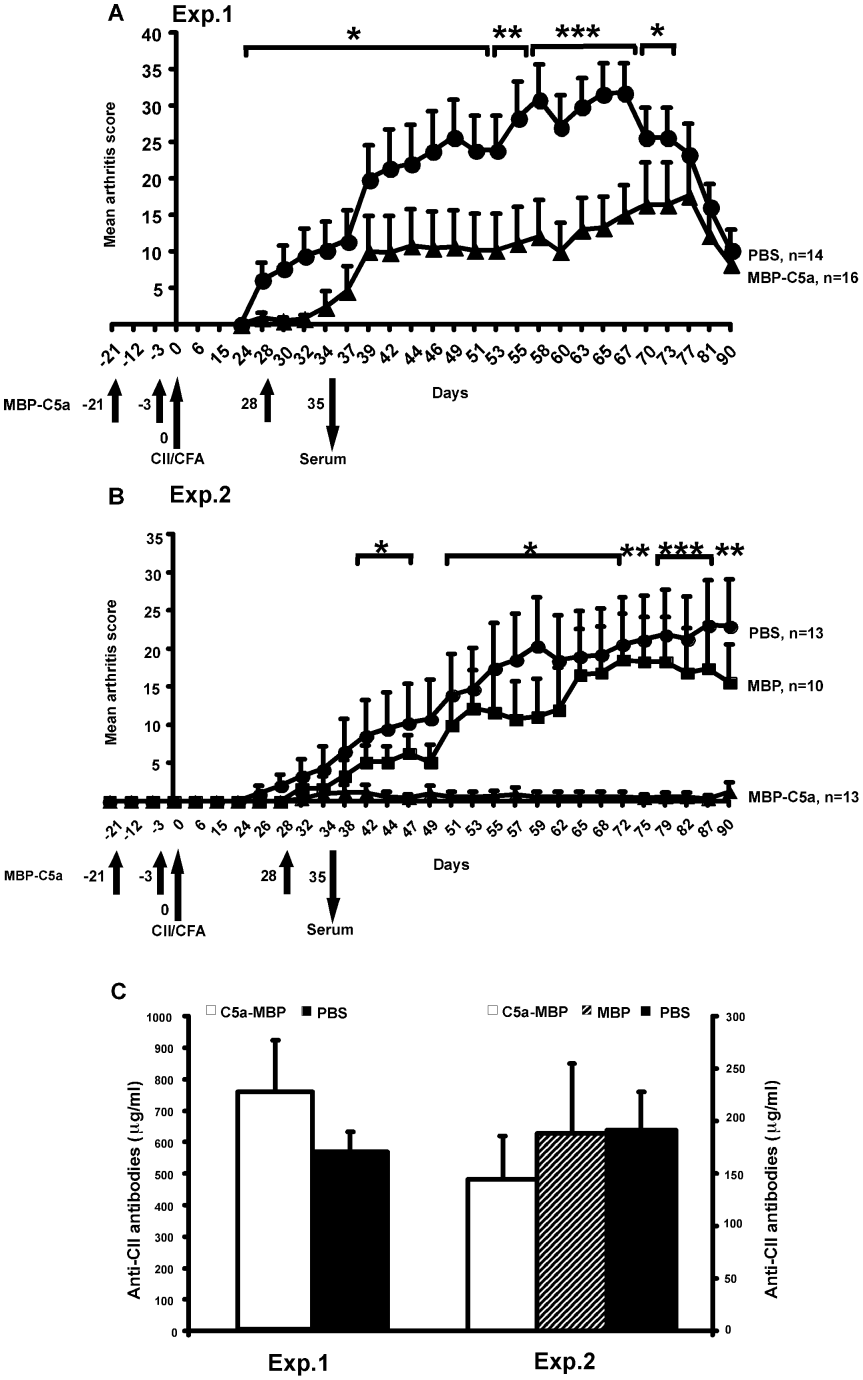
Inhibition of CIA by MBP-C5a vaccination. Mean clinical score of arthritis severity from two representative experiments; A. (BALB/c x B10.Q) F1 male mice (8 weeks old) received vaccination subcutaneously of 100 µg MBP-C5a or PBS emulsified in CFA on day −21 and were re-vaccinated on days −3 and +28 with 50 µg of MBP-C5a or PBS emulsified in IFA as indicated by arrows. B. Similar protocol as above including MBP group. In both the experiments, mice were immunized with 100 µg of rat CII in CFA on day 0. Serum samples were collected on days 0 and 35. All the animals were included for calculations and the data represent mean ± SEM. Significance of differences in severity of arthritis between MBP-C5a and PBS groups was analyzed by Mann–Whitney U ranking test. *, p<0.05; **, p<0.005 and ***, p<0.001. 100% and 44% of mice developed arthritis in PBS or MBP-C5a vaccinated groups in the first experiment. During the second experiment, 13%, 60% and 69% of the mice developed arthritis in MBP-C5a, MBP or PBS vaccinated groups. C) Anti-CII antibody levels in the above two experiments. No significant difference in CII-specific antibody levels was observed between groups. n, denotes number of mice used in each group.

**Figure 2:**
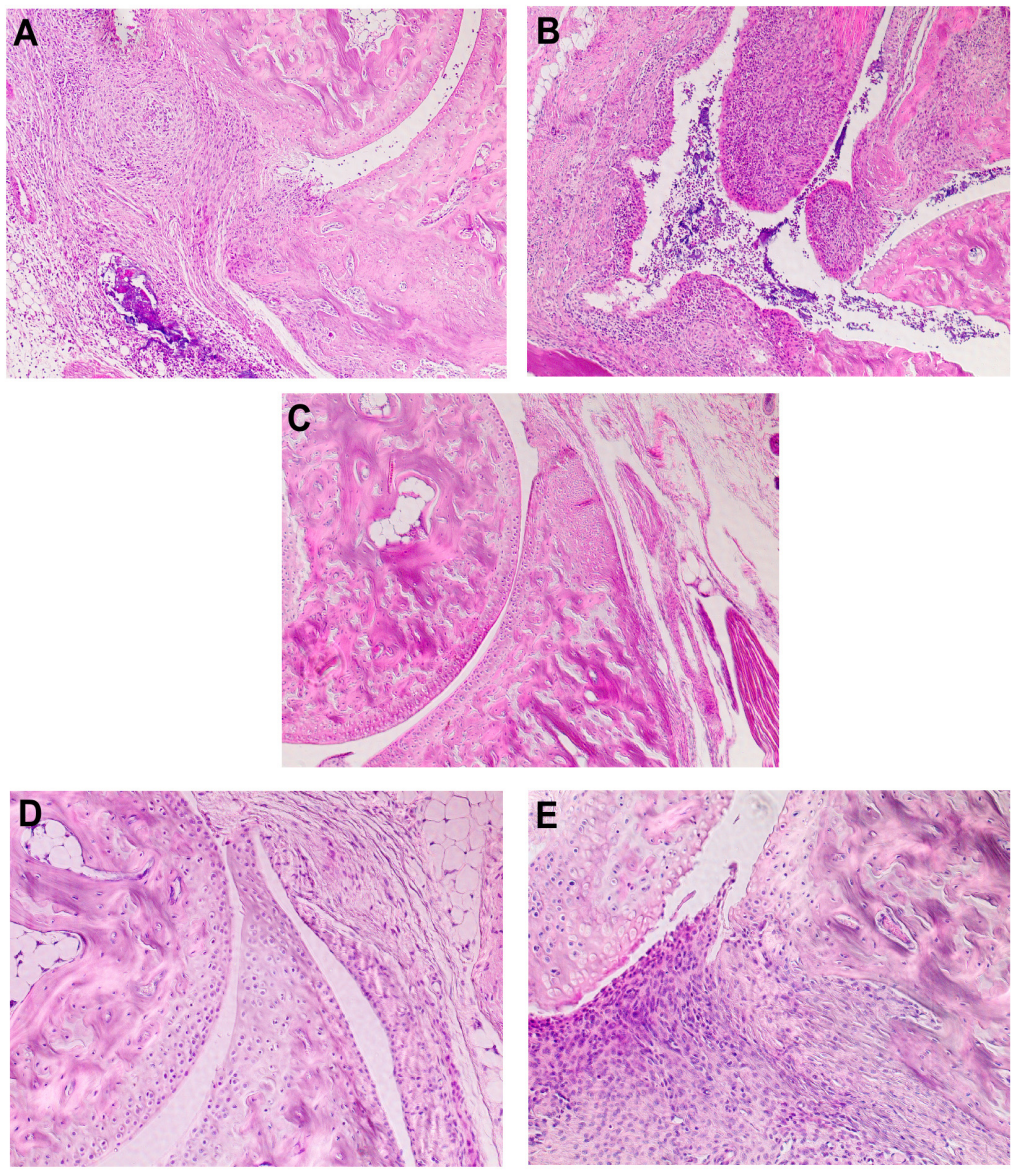
Histology of joint sections. Effect of C5a inhibition in CIA (A–C) and CAIA (D–E) demonstrated in representative tissue histology. 4–5 mice in each group from the experiments shown in Fig. 1B (CIA) and Fig. 3 (CAIA) were sacrificed and joint morphology was assessed using a standard haematoxylin/eosin staining protocol. Representative joint sections (6 µm) from QB mice induced with CIA but vaccinated with PBS (A), MBP (B) or MBP-C5a (C) are shown. Magnification x10. Joint sections from mice induced with CAIA and vaccinated with MBP-C5a (D) or control (PBS) (E) are shown. Magnification x20.

Since widely used animal models for RA are dependent on antibody-mediated pathologies and complement is one of the major effector mechanisms, we used CIA to test the vaccine potency of MBP-C5a. Two separate experiments were performed in male (BALB/c x B10.Q) F1 mice and arthritis was found to be significantly attenuated (Fig. 1A and B). Importantly, we did not find any significant difference in CII-specific antibody levels between groups (Fig. 1C). Histology of the joint sections of CIA mice vaccinated with PBS (Fig. 2A) or MBP (Fig. 2B) showed extensive cartilage and bone erosions with massive infiltration of cells. However, joints from MBP-C5a vaccinated CIA mice were without any significant cellular infiltration or cartilage and bone damage (Fig. 2C). Comparable vaccinating effect of MBP-C5a was observed in mice with another genetic background (B10.Q x DBA/1) F1 of both sexes and in (BALB/c x B10.Q) F1 female mice. Similar results were observed when MBP-C5a produced with a new linker was used in DBA/1 mice (data not shown).

### C5a vaccination significantly inhibited arthritis induced with anti-CII mAbs

**Figure 3:**
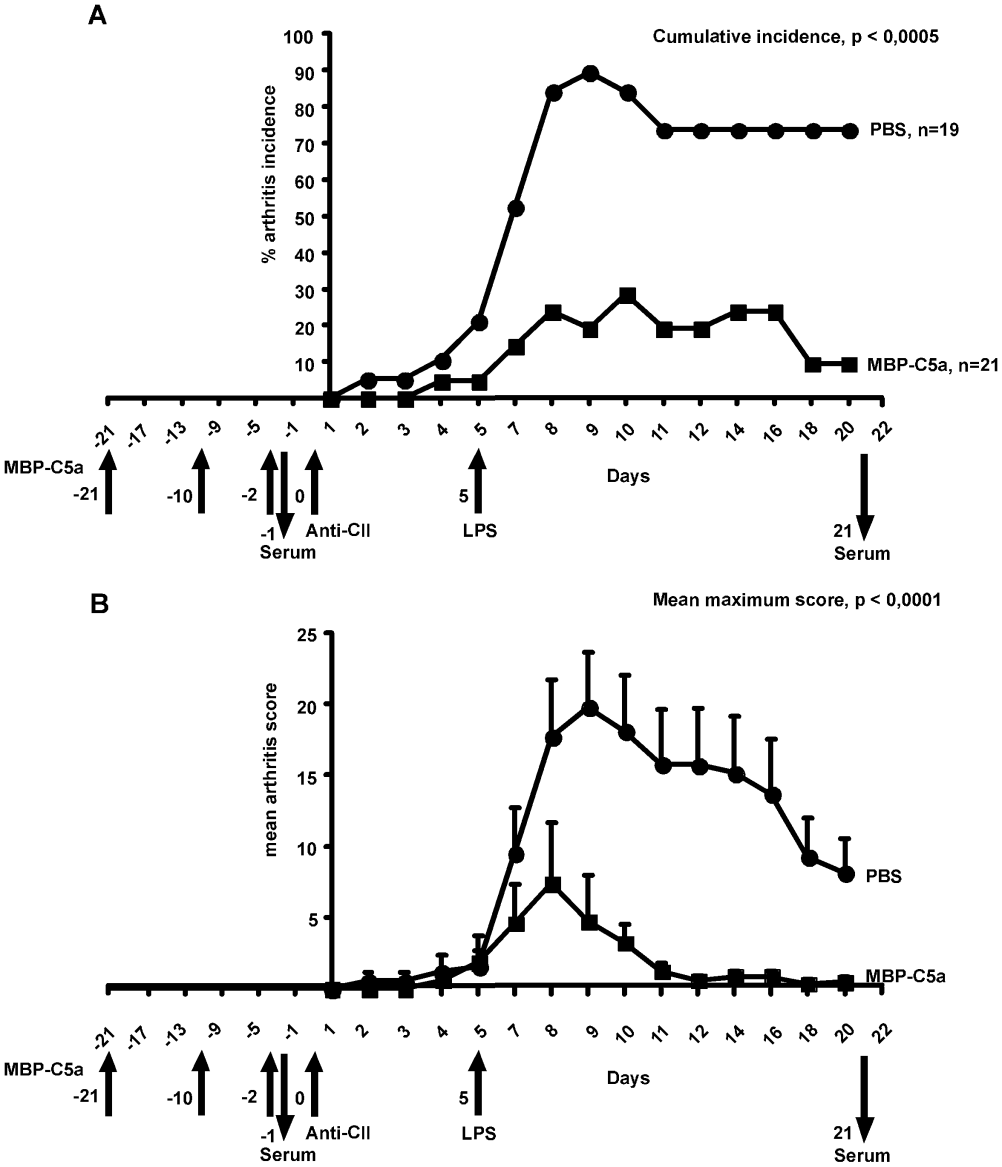
Effect of MBP-C5a vaccination in CAIA. Four-month-old male QB mice were injected intravenously with a cocktail of three complement activating monoclonal antibodies (6 mg in total/mouse) binding to CII on day 0, followed by LPS (25 µg/mouse intraperitoneally) on day +5. MBP-C5a or PBS emulsified in CFA/IFA was injected at the back of the mice on days −21, −10 and −2 as indicated by arrows. The mice were scored every day and the graphs present (A) incidence (maximal possible value is 100) and (B) severity of arthritis (maximal possible value is 60). Serum samples were collected from these mice on days −1 and +21 as indicated in the figure. Error bars indicate SEM and n denotes number of mice in each group. The severity of arthritis was analyzed by Mann–Whitney U ranking test and the incidence by χ^2^ test. C5a vaccinated animals had less frequent (38% versus 95%; p<0.0005) as well as severity of arthritis (mean maximum score, p<0.0001) compared to control (PBS with CFA or IFA) vaccinated animals. n, indicates number of mice in each group.

To ascertain the therapeutic potential of C5a vaccination at the effector phase of autoimmune diseases, we used CAIA model using a cocktail of IgG2b mAbs binding to 3 dominant B cell epitopes of CII and the C5a vaccinated animals had less frequent and reduced arthritis severity compared to control vaccinated animals (Fig. 3A and B). C5a vaccinated, anti-CII antibody injected mice showed neither significant cellular infiltration nor cartilage and bone erosions (Fig. 2D), whereas massive infiltration of immunocytes, pannus formation, fibrin deposition, and distinct bone and cartilage erosions were observed in the antibody-injected, control vaccinated mice (Fig. 2E).

### Level of C5a- specific antibodies and C5a inhibitory capacity

**Figure 4:**
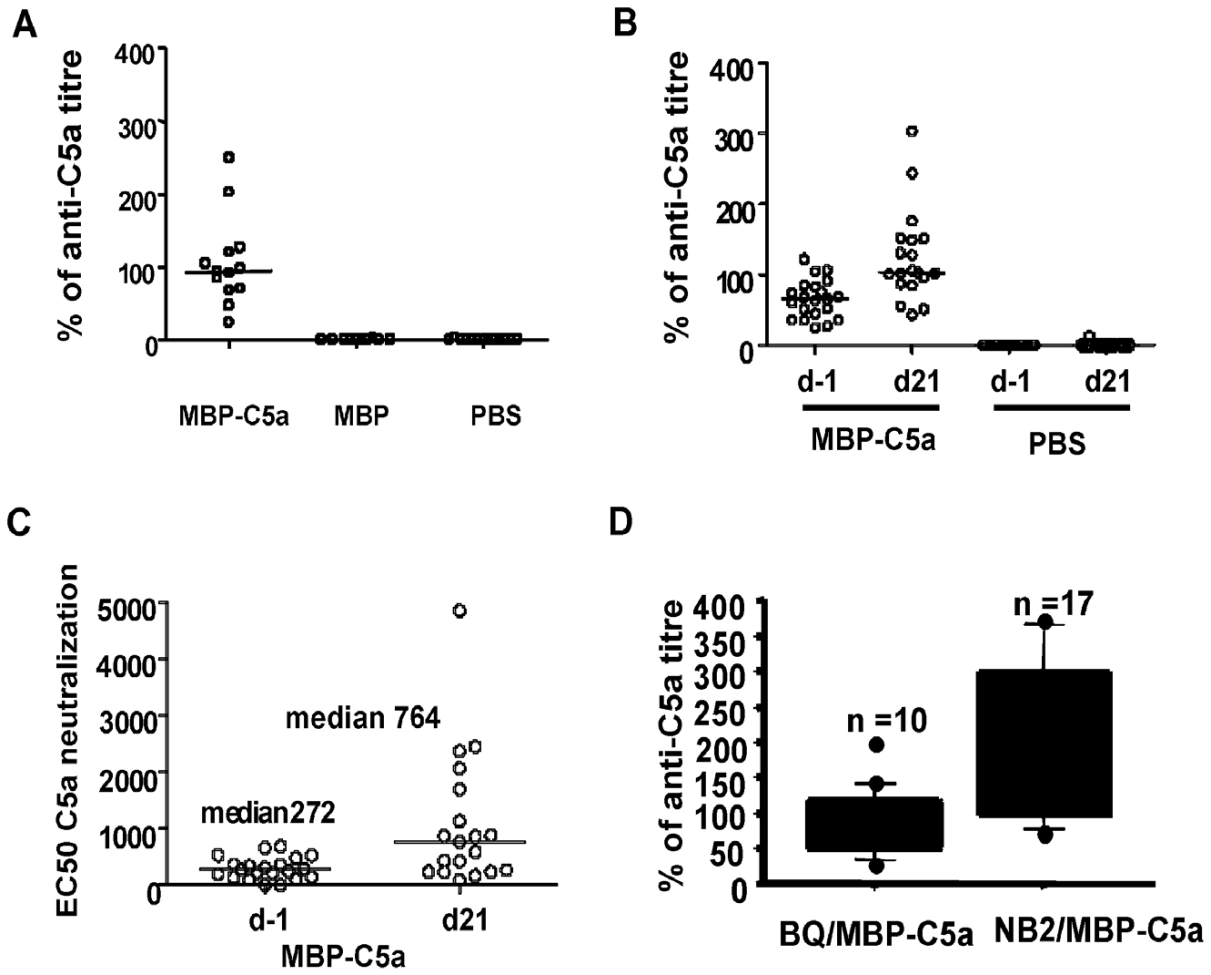
Determination of C5a-specific antibody levels and C5a neutralization. Anti-C5a antibody levels (A) in the sera collected from mice on day 35 immunized for CIA. As described in Fig. 1B, (BALB/c x B10.Q) F1 male mice (8 weeks old) received vaccination subcutaneously of 100 µg MBP-C5a or PBS emulsified in CFA at day −21 and were re-vaccinated on days −3 and +28 with 50 µg of MBP-C5a or PBS emulsified in IFA. All the mice were immunized with 100 µg of rat CII in CFA on day 0. Anti-C5a antibody levels (B) and C5a neutralization capacity (C) present in the sera collected from mice on day −1 and day 21. The mice were vaccinated with MBP-C5a or PBS emulsified in CFA/IFA on days −21, −10 and −2 and used in CAIA experiment on day 0, as shown in Fig. 3. Anti-C5a antibody levels (D) present on day 35 in B10.Q mice and the C5 deficient congenic mice strain B10.Q.NOD-*Cia2* (NB2). Groups of 8 weeks old mice received vaccination subcutaneously of 100 µg MBP-C5a or PBS emulsified in CFA on day −21 and were re-vaccinated on days −3 and +28 with 50 µg of MBP-C5a or PBS emulsified in IFA. Upon vaccination with MBP-C5a protein, C5 deficient mice (NB2) responded with higher anti-C5a levels than C5 sufficient B10.Q mice (p = 0.018). n, indicates number of mice in each group. All the assays were done in triplicates.

All individuals receiving C5a but not control vaccinations developed anti-C5a IgG antibodies (Fig. 4A and B). Subsequently, the ability of serum from C5a vaccinated mice to inhibit C5a-mediated degranulation was determined using a RBL cell line transfected with human C5aR. The amount of released β-hexosaminidase was directly proportional to the amount of C5a added within the range of 2000 ng/ml (240 ng per well) to 2.7 ng/ml (0.3 ng per well). Serial dilutions of serum were added to 5.6 ng of C5a and the degree of inhibition was determined. All the animals receiving C5a but not control vaccination displayed C5a inhibitory capacity (Fig. 4C). Upon vaccination with MBP-C5a protein, C5 deficient mice responded with higher anti-C5a levels than C5 sufficient mice (p = 0.018) and anti-C5a antibodies can be induced in the vaccinated C5 sufficient mice (Fig. 4D).

### Inhibition by C5a vaccination of CII-induced relapse of chronic CIA

**Figure 5:**
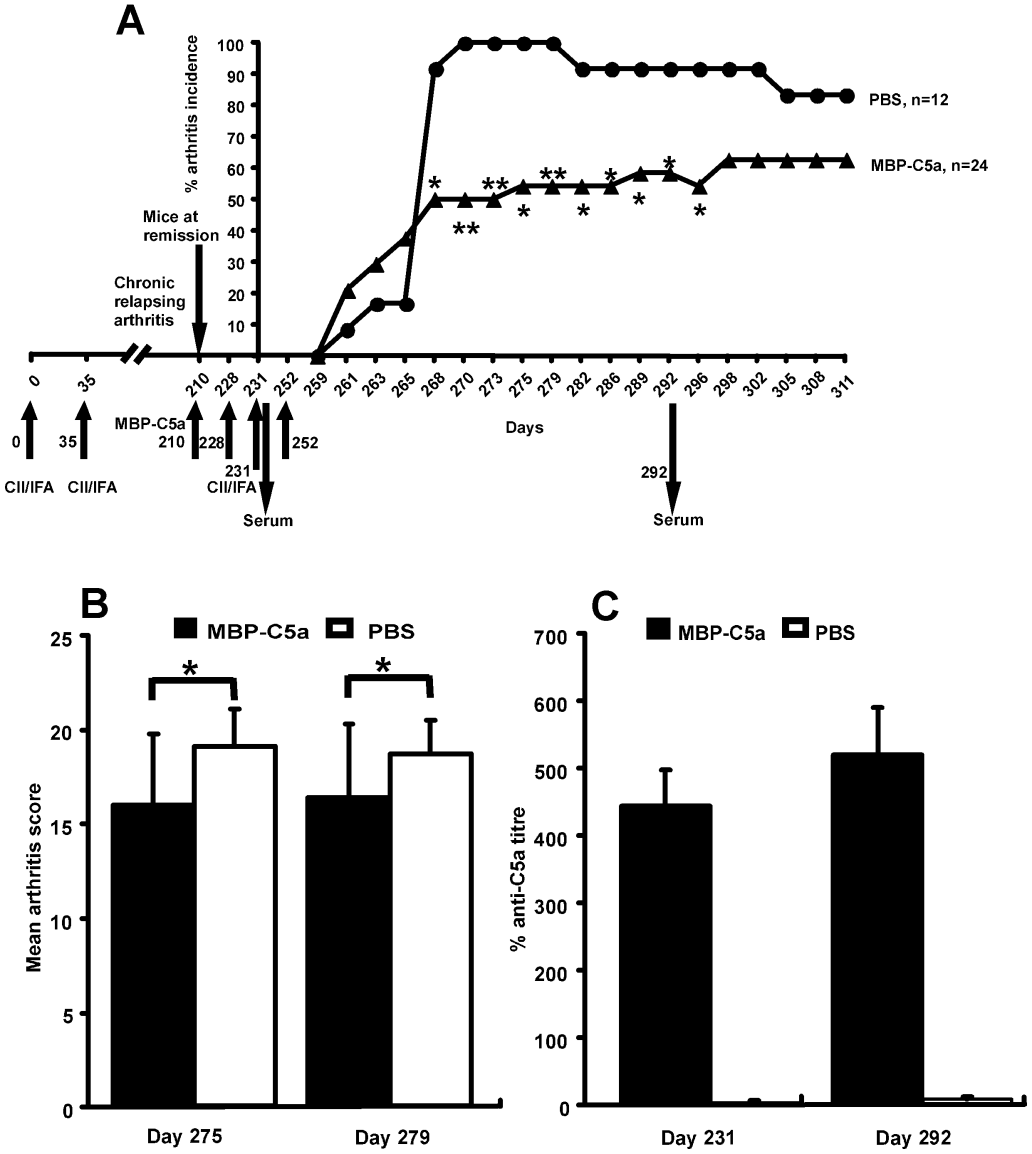
Effect of MBP-C5a vaccination on chronic arthritis. Frequency (A), mean arthritis score (B) and levels of C5a-specific antibodies (C) were compared between MBP-C5a and PBS vaccinated groups. B10.Q (BALB/c x B10.Q)F_2_ mice of both gender (8 weeks old) were immunized with 100 µg of rat CII emulsified in IFA on day 0 at the base of the tail and boosted on day 35 with 50 µg of rat CII in IFA as indicated by arrows. The mice were scored for a period of 210 days for arthritis development. Mice that developed chronic arthritis and at arthritis remission were selected for the treatment experiment. All selected animals received vaccination subcutaneously of 100 µg of MBP-C5a or PBS emulsified in CFA on day 210 and were re-vaccinated on days 228 and 252 with 50 µg of MBP-C5a or PBS emulsified in IFA s.c. at the back. All the animals were re-immunized for synchronization of arthritis induction on day 231 with 50 µg of rat CII in IFA and scored the next 80 days for clinical signs of arthritis up to day 311 (from first day of CII immunization). Serum samples were collected on day 231 and 292. Statistical analysis for mean arthritis score was done on all days (d259 to d311) but the significance between groups was found only on days 275 and 279. All data represent mean ± SEM. *, p<0.05 and **, p<0.005. n, indicates number of mice in each group. All the mice were included for calculation of arthritis susceptibility and severity.

To investigate whether MBP-C5a vaccination could be efficacious in the treatment of chronic arthritis, we immunized B10.Q (BALB/c × B10.Q) F_2_ mice with rat CII in IFA, which develops chronic relapsing arthritis [19]. These are genetically heterogeneous mice, and only mice with severe and active chronic relapsing arthritis for a minimum period of 120 days but at arthritis remission at day 210 were selected. For synchronization of relapse, we reimmunized them with CII emulsified in IFA on day 231. The mice were scored upto day 311 (Fig. 5A). All the mice developed arthritis in the control group, whereas a significant decrease in the incidence of arthritis (Fig. 5B) was observed in MBP-C5a vaccinated group. However, severity of arthritis was significantly reduced only on two different days (275 and 279), when all the mice were included for calculations. On the other hand, when we analyzed significance in severity of arthritis between the groups using sick mice only, the arthritis severity tended to be lower in the C5a treated group but reached significant difference only on day 265 (p = 0.0330). High levels of C5a-specific neutralizing antibodies could be detected in the C5a but not control vaccinated mice (Fig. 5C).

### Vaccination did not affect activation of C5 and activity of C5b

**Figure 6:**
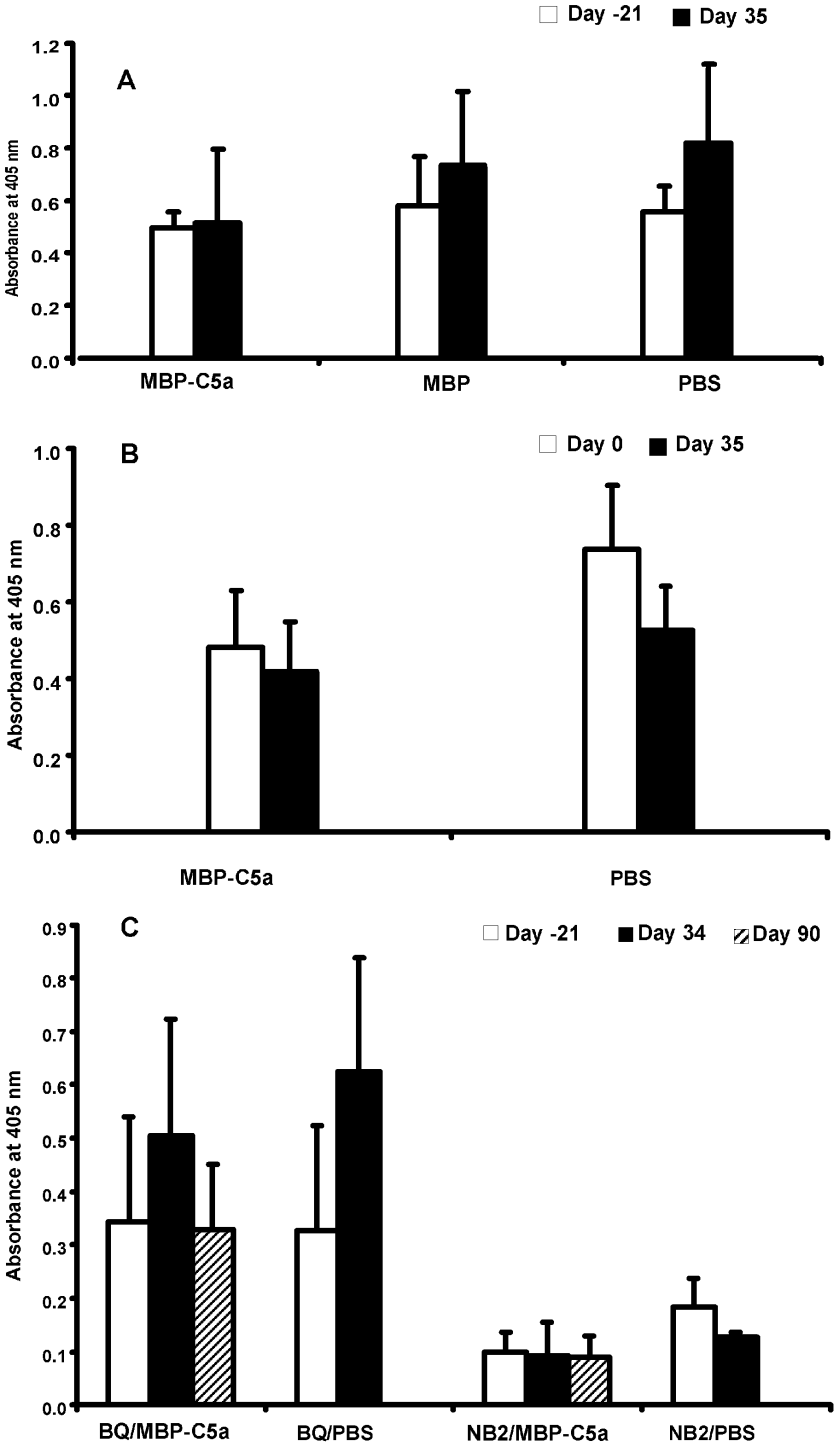
Measurement of C5 activity in mouse sera. Sera were collected on indicated days from mice described in Fig. 1B (CIA), Fig. 3 (CAIA) and Fig. 4D (C5 sufficient B10.Q and C5 deficient NB2 mice). Serum samples were analyzed for C5 activity A) on days −21 and 35 from MBP-C5a, MBP or PBS vaccinated mice used in CIA experiment, B) day 0 and 35 from MBP-C5a or PBS vaccinated mice used in CAIA experiment and C) day −21, 34 and 90 from MBP-C5a or PBS vaccinated C5 sufficient B10.Q or C5 deficient NB2 congenic mice. Great care was taken in order to preserve complement activity. Activity of the alternative pathway of complement was measured in hemolytic assay in which rabbit erythrocytes were incubated with mouse sera and the amount of hemoglobin released in supernatants due to lysis was determined spectrophotometrically. All the assays were done in triplicates. There was no significant difference in complement activity between MBP-C5a and MBP control (A) but there was a significant difference in complement activity when MBP-C5a and PBS groups were compared (in Fig. A and B but not in C) using t-test (paired). Error bars indicate ± SD.

Since C5b has important functions independent of C5a, we investigated if vaccination affected the degree of activation of C5 and function of C5b. Sera of mice vaccinated with C5a or control showed identical hemolytic activity in CIA experiment 2 (Fig. 6A), CAIA (Fig. 6B) and B10.Q mice (Fig. 6C). Hemolytic assay measures the whole alternative pathway of complement including assembly of MAC complex, which includes C5b. Therefore, we conclude that vaccination against C5a did not affect cleavage of C5 significantly, i.e. generation of C5b as well as the ability of C5b to become incorporated into MAC.

## Discussion

Targeting complement system is a promising strategy in drug discovery for treating inflammatory diseases [20]–[22]. Here, we developed an immunotherapeutic strategy based on the principle of introducing an active ingredient that stimulates the production of antibodies against C5a. The immunotherapeutic fusion protein (MBP-C5a) developed targets C5a by inducing a polyclonal antibody response. The pharmacologically active domain (C5a) is derived from the species to be treated, whereas the fusion protein is a bacterial protein fused to the N-terminus. Using this strategy, we produced the fusion proteins using mammalian expression vectors and evaluated their vaccinating potential. Neutralizing antibodies induced upon immunization were specific to C5a and they did not alter C5/C5b activity significantly but led to attenuation of arthritis in various mouse models. Furthermore, both C5 deficient and sufficient mice developed anti-C5a response but the former group responded comparatively stronger, which demonstrate the presence of tolerance towards C5a and the possibility of breaking such tolerance by this vaccination procedure. Importantly, generation of C5a-specific antibodies did not disturb the normal C5 function.

Antibodies against self proteins are prevalent in arthritis, which in the form of immune complexes on or within the joint cartilage can trigger complement activation and recruitment of FcγR+ neutrophils leading to release of cytokines, chemokines, destructive proteases and oxidants [15], [17]. In antibody induced arthritis, though both the classical and alternative pathway of complement activation are essential [23], contribution of the alternative pathway was well emphasized [24], [25]. Furthermore, apoptotic and necrotic cells that may be present in affected joints could also activate complement [26] and so do some cartilage proteins that become exposed during cartilage damage such as fibromodulin [27].

Activation of complement cascade leads to the formation of C5 convertases, generating C5a and C5b from C5 but complement-independent enzymes, such as thrombin [28], neutrophil elastase [29] and a macrophage serine protease [30] can also generate C5a. Importantly, C5a thus generated is one of the most potent inflammatory peptides involved in the recruitment and activation of inflammatory cells, release of granule-based enzymes and enhancing cell adhesion thereby inducing release of various inflammatory mediators such as histamine or cytokines all of which may contribute to innate immune functions or tissue damage [9], [31], [32]. Furthermore, C5a can act as a general regulator of FcR expression [33] and FcγR signaling can also be involved in the generation of C5a [34]. A single two nucleotide difference in the FcγRIII and IIb gene promoters was found to determine their inverse responsiveness to C5a [35]. Interestingly, C5aR antagonist was effective in treating sepsis [36], reducing antigen-induced arthritis [37] and neurodegeneration [38]. However, blocking of C5aR in small cohort of RA patients (n = 21) did not reduce synovial inflammation [39] and the orally administered C5aR-antagonist AcF- (OpdChaWR) (PMX53) in that study binds to C5aR but not C5L2 [40]. Since C5L2 is uncoupled from G proteins, it has been suggested that this receptor acts as a decoy receptor that regulates the activity of C5a and C5a desArg. But a recent study on experimental sepsis describes a pro-inflammatory role for C5L2, which contributes to the release of mediators in the inflammatory response [41]. This becomes especially important because C5L2 is required for the release of high mobility group box 1 protein (HMGB1) [41] that has been shown to have an important role in the pathogenesis of arthritis [42]. Furthermore, since we observed significant reduction in CAIA induced only with complement fixing antibodies, another possibility for the inefficiency of C5aR blockade might be attributed to the presence of non-complement fixing antibody subclasses in RA patients but this information is not available for that study [39]. Interestingly, vaccination with C5a could very well be useful in other clinical conditions since neutralization of C5a led to amelioration of pathological changes developed in sepsis [43] and burn-induced cardiac dysfunction [44]. Similarly, genetic ablation of C5aR is very effective in preventing inflammation in CAIA [32] and anti-G6PI dependent arthritis [45]. Anti-human C5aR antibodies prevented arthritis in human C5aR knock-in mice [46]. Recent studies also showed generation of C5a *in vitro* by anti-CII mAb from mouse sera [25].

Importantly, a specific inhibition of C5a biological activity, without affecting the beneficial effects of C5/C5b, could gain therapeutic benefit without affecting the protective immune responses [47]. Specific blockade of C5 cleavage thereby preventing C5a generation was achieved either by a humanized mAb or its single chain [48]. However, studies done with anti-C5 antibodies (Eculizumab) in arthritis gave mixed results and were published only as web release. Other way of neutralizing the C5a function is by using pharmacological inhibitors or antibodies apart from C5a receptor (s) targeting inhibitors. Here, we could demonstrate the feasibility of generating anti-C5a antibodies by the host after immunization with C5a fusion protein that did not compromise C5/C5b activity significantly but led to attenuation of arthritis in various mouse models. Thus, it is possible to break tolerance against the self-protein C5a using MBP-C5a vaccination as evidenced by the high anti-C5a antibody response. The advantage of this method is to exploit the host immunity for its own benefit as well as avoiding neutalization of the injected mAb or inhibitors by the host. An obvious risk with a vaccination approach is that it could be difficult to reverse in case of side effects. Although more work is needed on this direction, we think it is possible to handle; firstly the increased anti- C5a titers are quite shortlasting and there are technologies available now for cleaving antibody activities *in vivo* [49]. Although none of the mice injected with the MBP-C5a vaccine developed any infectious complications, we can not rule out the increased risk of intracellular infections because of the neutralization of C5a [10]. Thus, complement inhibition as a therapeutic treatment could be achieved by interventions aimed at blocking the pathological activities of complement activation products, while avoiding disruption of the role of complement in host immunity. Thus, this approach is more specific than depleting C5 and since the production of antibodies occur at the site of inflammation it could be a treatment that has limited risk of side effects. However, obviously more studies are needed before using this strategy for clinical trials.

## Materials and Methods

### Ethics statement

All experiments were performed on age-matched mice between the ages of 8 and 16 weeks with the approval of animal ethics committee, Malmö-Lund region, Sweden.

### Mice

Breeding pairs of BALB/cJ and B10.Q mice were from Jackson Laboratories (Bar Harbor, ME) and Prof. Jan Klein (Professor Emeritus, Tübingen University, Tübingen, Germany) respectively. The congenic B10.Q.NOD-*Cia2* mice used were C5 deficient [50].

### Construction, expression and purification of recombinant MBP-C5a

Mouse liver Poly A+ RNA (Clontech Laboratories Inc., CA) was used for amplifying cDNA encoding C5a by One-Step RT-PCR (Qiagen Inc., CA) using forward primer Va5′ (CGCCAGCTGCTAAGGCAGAAAATAGAAG) and reverse primer Va3′ (CGCGTCGACTTACCTTCCCAGTTGGACAGG). MBP-C5a was amplified using forward primer 816 (CCAGAATTCCACCATCACCATCACCATCTCGAGCCGCGGG CCGATATGAAAATCGAAGAAGG) and reverse primer Va3′. Plasmid DNA was prepared using Qiagen Midiprep kit and EBNA-293 cells were transfected using lipofectamine 2000 (Invitrogen AB, Sweden). Cells were expanded and supernatants were collected for purification using ion exchange (SP Sepharose FF) and affinity (amylose) chromatography. SDS-PAGE, size exclusion chromatography and western blot were used to determine the purity of the proteins (data not shown).

### Detection of anti-C5a antibodies in mouse serum

Serum IgG anti-C5a antibodies were determined by a semi-quantitative ELISA. Briefly, samples were titrated in five-fold dilutions similar to reference serum in ELISA plates coated with C5a (4 µg/ml). Alkaline phosphatase-conjugated goat anti-mouse IgG Fcγ-specific antibody (Jackson ImmunoResearch, PA) and p-nitrophenyl phosphatase and buffer tablets (Sigma-Aldrich, MO) were used as detection system. The absorbance was read at 405 nm. The titer (the dilution yielding a 50% reduction of absorbance) was determined for each sample and the reference serum. The titer of each sample was related to the reference serum and this was termed as relative titer (%).

### C5a neutralization assay

A rat basophilic leukemia (RBL) cell-line transfected with human C5aR was used. Upon binding of C5a to the receptor, N-acetyl-β-D-glucosaminidase (NAG) is released, which is quantified by addition of its substrate. Serum containing antibodies that inhibits this binding will result in a decreased absorbance. At first, RBL cells (2×10^6^ cells/ml) were incubated 3 minutes in 37°C with cytochalasin B (Sigma-Aldrich) to enhance C5a-induced enzyme release. Serum samples were incubated in HBSS medium containing 2 mM HEPES, 0.2% BSA and 40 µg/ml DL-2-mecaptmethyl-3-guanidinoethylthiopropanoic acid (Calbiochem) for one hour at 37°C, followed by addition of mouse C5a (HyCult Biotechnology) at 35 ng/ml and incubation. As an internal control, C5a was incubated with HBSS medium. Triton X-100 (0.2%) was added as a control for maximal enzyme release. Plain medium and a reference serum were used as negative and positive controls respectively. Eighty µl of the RBL cell suspension was thereafter exposed to the serum containing C5a for 6 min at 37°C. To stop the reaction, the plates were centrifuged for 3 min, 1000× g, at 4°C. Seventy µl of the supernatant was transferred to a 96 well plate with 70 µl of substrate solution [7.5 mM of p-nitrophenyl N-acetyl-β-D-glucosaminide (Sigma-Aldrich) dissolved in 42.5 mM sodium acetate buffer, pH 4.5] and incubated at 37°C for 3 hours. After incubation, 70 µl of glycine buffer (0.2 M, pH 10.7) was added and the absorbance was read at 405 nm.

### Induction and vaccination of collagen induced arthritis – classical and chronic relapse

(BALB/c x B10.Q mice) F1 mice were vaccinated with 100 µg of MBP-C5a, MBP or PBS emulsified in CFA, s.c. on day -21 and re-vaccinated on days -3 and +28 with 50 µg of MBP-C5a, MBP or PBS emulsified in IFA s.c. For induction of arthritis, mice were immunized with 100 µg of rat CII (rCII), emulsified 1∶1 in CFA (Difco, Detroit, MI) on day 0. Mice were monitored for arthritis on the indicated days and serum samples were collected on days 0 and 35.

For induction of chronic relapsing arthritis, B10.Q (BALB/c x B10.Q)F_2_ mice were immunized with 100 µg of rCII emulsified in IFA on day 0 and boosted on day 35 with 50 µg of rCII in IFA. Mice that developed chronic arthritis (defined as severe arthritis for a minimum of 120 days) but exhibited no active arthritis at day 210 were selected for treatment. All selected animals received vaccination of 100 µg of MBP-C5a or PBS emulsified in CFA, s.c. on day 210 and re-vaccination on days 228 and 252 with 50 µg of MBP-C5a or PBS emulsified in IFA s.c. All the animals were reimmunized for synchronization of arthritis on day 231 with 50 µg of rCII in IFA and scored upto day 311 (from first day of CII immunization). Serum samples were collected on days 231 and 292.

### Induction and vaccination of collagen antibody induced arthritis (CAIA)

B cell hybridomas specific to CII were generated and characterized previously [51], [52]. Antibodies from clone M2139 (IgG2b) bind J1 epitopes of CII (551–564; GERGAAGIAGPK), CIIC2 (IgG2b) bind D3 epitopes of CII (687–698; RGAQGPPGATGF), and UL1 (IgG2b) bind U1 epitopes of CII (494–504; GLVGPRGERGF). Groups of mice were vaccinated with 100 µg of MBP-C5a or PBS emulsified in CFA, s.c. on day −21 and revaccinated on days −10 and −2 with 50 µg of MBP-C5a or PBS emulsified in IFA s.c. A cocktail of the 3 mAbs (6 mg per mouse) was injected i.v. and on day 5, LPS (25 µg/mice) was injected i.p. to enhance incidence and severity of arthritis.

### Clinical evaluation of arthritis and histology

Scoring of animals was done blindly using a scoring system based on the number of inflamed joints in each paw, inflammation being defined by swelling and redness [53]. In this scoring system each inflamed toe or knuckle gives one point, whereas an inflamed wrist or ankle gives five points, resulting in a score of 0–15 (5 toes+5 knuckles+1 wrist/ankle) for each paw and 0–60 points for each mouse. Paws from each group of mice (4–5 per group) were dissected, fixed, decalcified, dehydrated, and embedded in paraffin. Sections (6 µm) were stained with hematoxylin-eosin.

### Anti-CII antibody response

The amounts of total anti-CII IgG were determined using quantitative ELISA [54]. Sera from non-immunized syngenic mice were used as a negative control and did not contain any detectable amounts of anti-CII Abs. Affinity purified anti-CII antibody from pooled sera of CII immunized mice was used as standard.

### Complement analysis

Complement activity was determined using a hemolytic assay. Rabbit erythrocytes were washed in Mg-EGTA buffer (2.5 mM veronal buffer pH 7.3, 70 mM NaCl, 140 mM glucose, 0.1% gelatin, 7 mM MgCl_2_ and 10 mM EGTA) and resuspended at 0.5×10^9^ cells/ml. Mouse serum (5 µl) was incubated for 3 h at 37°C with 20 µl of erythrocyte suspension in 100 µl of Mg-EGTA buffer and the degree of lysis was determined by the amount of released hemoglobin measured at 405 nm and the absorbance provided by serum itself was subtracted from the values. Low level of C5 activity in C5 deficient mice (Fig. 6C) also served as the negative control for C5 assays.

### Statistical analyses

All the mice were included for calculation of arthritis susceptibility and severity. Severity and incidence of arthritis was analyzed by Mann Whitney U test and Chi Square analysis respectively using Statview 5.0.1 (SAS Institute, NC). Significance was considered when p<0.05.
